# Age Differences in Flow Experience During Body Movement–Controlled Video Game Rehabilitation Tasks: Cross-Sectional Study

**DOI:** 10.2196/76278

**Published:** 2025-10-29

**Authors:** Shiyi Tong, Lixuan Li, Yuankun Zhang, Weiyan Ren, Fang Pu

**Affiliations:** 1 School of Biological Science and Medical Engineering, Beihang University, 37 Xueyuan Road, Haidian District, Beijing, 100191, China, 86 13521079466; 2 Department of Medical Innovation and Research, Chinese PLA General Hospital, Beijing, China; 3 School of Engineering Medicine, Beihang University, Beijing, China

**Keywords:** flow, rehabilitation, video games, exergames, task design, age differences

## Abstract

**Background:**

Body movement–controlled video games (BMCVGs) are increasingly adopted in rehabilitation because they combine physical training with interactive engagement. Flow experience, a critical factor for enhancing user engagement and training efficacy, exhibits age-related differences that are essential for designing age-appropriate rehabilitation tasks. However, current BMCVG rehabilitation tasks often overlook these age-related differences in subjective experience, leading to insufficient engagement among older adults.

**Objective:**

This study aimed to explore differences in flow experience between younger and older adults when performing the same BMCVG rehabilitation task and to provide empirical evidence for designing personalized and age-appropriate programs.

**Methods:**

A total of 40 participants were recruited, including 21 older adults (mean age 63.00, SD 6.64 y; n=10, 48% male participants) and 19 younger adults (mean age 24.68, SD 1.16 y; n=9, 47% male participants). Participants performed the “Space Pop” task in *Kinect Adventures*, simulating limb coordination training. Flow experience was assessed using the Chinese version of the Flow State Scale-2, which measures 9 dimensions of flow. Group differences were analyzed using the nonparametric Wilcoxon rank-sum tests, and effect sizes (Cohen *d*) were calculated via bootstrap estimation.

**Results:**

Older adults exhibited significantly lower overall flow experience than younger adults (W=339.5; *P*<.001; Cohen *d*=1.45; η^2^=0.37). Significant differences were also found in the dimensions of “challenge-skill balance” (W=339; *P*<.001); “clear goals” (W=271; *P*=.04); “sense of control” (W=389.5; *P*<.001); and “loss of self-consciousness” (W=268; *P*=.048). The largest effect was observed in the “sense of control” dimension (Cohen *d*=3.22; η^2^=0.74), indicating it was the most significantly impacted by age. Other dimensions (eg, concentration and time transformation) showed no significant differences.

**Conclusions:**

Age plays a significant role in shaping flow experiences during BMCVG rehabilitation tasks. Older adults’ reduced flow may be attributed to declines in cognitive processing speed, motor control, and self-efficacy, which particularly impair their sense of control and goal clarity. Tailoring designs through strategies such as dynamic difficulty adjustment, clearer goal cues, and reduced motor demands is crucial. These adaptations can enhance older adults’ sense of control and immersion, promoting active participation and ultimately improving rehabilitation outcomes.

## Introduction

### Role of Flow Experience in Body Movement–Controlled Video Games for Rehabilitation

“Flow” is a state of optimal experience where individuals are fully engaged in challenging activities, experiencing high focus, control, and enjoyment [1]. Due to its positive effects, flow has been widely studied in sports, music, gaming, learning, and other fields [2-5], enhancing performance, engagement, and task persistence.

In the context of body movement–controlled video games (BMCVGs) applied to rehabilitation, the characteristics of flow are particularly important. BMCVGs are a genre of video gaming where users control the game through their own body movements [6,7]. These games, which also belong to exergames, are increasingly being explored for their potential in rehabilitation, such as fall prevention and improving balance and mobility [8,9]. However, despite advancements in BMCVG hardware, rehabilitation task design often fails to integrate mechanisms that sustain engagement, leading to poor subjective experiences [10]. Current tasks often use a “one-size-fits-all” design, ignoring age-related differences in subjective experiences caused by older adults’ cognitive and motor ability declines, which affects training compliance. Flow theory provides a framework for assessing and optimizing user experiences, making it a useful tool for the design of BMCVG tasks intended for rehabilitation [11].

### Theoretical Basis for Age-Related Flow Differences

Flow is influenced by individual differences, including personality traits, gender, motivation, and mental health [2,12-15]. Research has shown that video game characteristics and player identification are important precursors to the flow state [6,16-18]. Age, a critical factor in psychological experiences, may influence flow in BMCVGs. Murman [19] noted that cognitive abilities (eg, information processing speed and executive function) decline with age, and Gazzaley and Nobre [20] emphasized older adults’ difficulties in attention allocation and maintenance. Boot et al [21] found that older adults have less video game experience and different preferences from younger individuals. These cognitive, attentional, and preference changes may impact their flow experience in BMCVG tasks. Older adults may face challenges in achieving flow due to cognitive and motor declines. However, empirical research on age differences in the flow experience within BMCVG rehabilitation remains limited, especially for typical rehabilitation tasks such as limb coordination training.

### Research Objectives and Hypotheses

This study compared 9D flow experiences between young and older adults in a BMCVG task that simulates activities often targeted in rehabilitation to test the hypothesis that age differences affect flow experience, aiming to (1) identify key dimensions of age-related flow experience differences and (2) provide theoretical evidence for adapting task difficulty and optimizing interfaces for older adults, promoting better design of BMCVG tasks for rehabilitation.

## Methods

### Participants

In total, 40 participants were recruited and divided into an older adult group (21 participants; mean age 63.00, SD 6.64 y; n=10, 48% male participants) and a young adult group (19 participants; mean age 24.68, SD 1.16 y; n=9, 47% male participants). Older adults were recruited from the community of Beihang University, while younger adults were students at Beihang University. Data collection took place between October and December 2024.

The inclusion criteria were as follows: (1) normal or corrected-to-normal vision, (2) no neuromuscular diseases, (3) no previous experience with Kinect, and (4) the ability to complete questionnaires independently. The exclusion criteria were as follows: (1) a current or historical medical recommendation against strenuous exercise and (2) any chronic illnesses that could impact physical or cognitive performance.

### Ethical Considerations

The study was approved by the Science and Ethics Committee of the School of Engineering Medicine, Beihang University (BM20230154). All participants were briefed on the study purposes and procedures, and they all gave written informed consent before participation. All collected data were deidentified by assigning unique participant ID codes in place of names. Each study participant was thanked and received 150 RMB (US $21) as a token of gratitude.

### Experimental Tools

The experimental equipment included an Xbox 360 (Microsoft Corp) gaming console, a Kinect v1 sensor (Microsoft Corp), and a 28-inch 4K display (Samsung U28E590D). The task selected for this study was “Space Pop” from Kinect Adventures (Microsoft Studios, Microsoft Corp), a BMCVG chosen because it involves skills pertinent to rehabilitation goals. The rehabilitation task selected was “Space Pop” from Kinect Adventures, which required participants to control a virtual character to pop bubbles through limb movements (forward, backward, left, and right) and arm actions (flapping and dropping). Real-time scores and ranks (none, bronze, silver, gold, and platinum) were displayed, with the task goal of popping as many bubbles as possible to simulate limb coordination and reaction speed training in rehabilitation.

The Flow State Scale-2 (FSS-2; Multimedia Appendix 1) [22] was adapted into the Chinese version (CFSS-2) through translation and back-translation. The scale consists of 9 dimensions (36 items). Participants completed a paper version of the questionnaire using a 5-point Likert scale (1=strongly disagree and 5=strongly agree). Instructions emphasized that there were no right or wrong answers.

### Experimental Procedure

**Figure 1.**
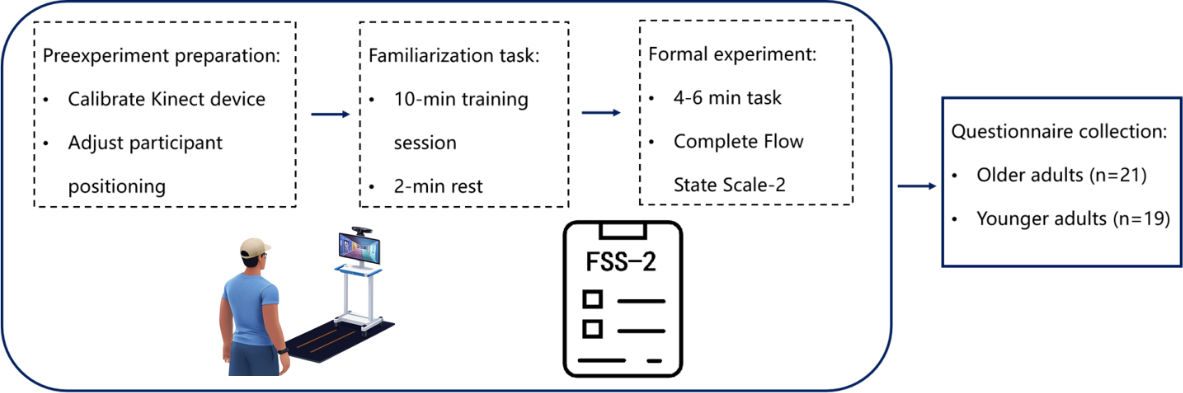
Experimental procedure for the body movement–controlled video game rehabilitation study using Kinect “space pop” task (Beihang University, Beijing, October-December 2024; 21/40, 53% older adults and 19/40, 47% younger adults).

Before the experiment, the Kinect sensor was calibrated for optimal recognition, and participant positioning was adjusted to the recommended distance (2‐3 m from the display) within the Kinect’s effective range. Participants completed a 10-minute training session (repeating the task twice) to ensure familiarity with the task operations, followed by a 2-minute rest to restore physical stamina. The formal task lasted 4‐6 minutes. After task completion, participants immediately completed the CFSS-2 questionnaire based on their experience during the task. The experiment was terminated immediately if any participant reported physical discomfort. The experimental procedure is outlined in Figure 1.

### Statistical Analysis

For each questionnaire, the mean score of the 4 items in each dimension was calculated as the dimension score and the mean score of all items in the questionnaire was calculated as the participant’s flow experience score during the task.

Using R (version 4.3.3; R Foundation for Statistical Computing), reliability and validity tests of the CFSS-2 were conducted by calculating the Cronbach α coefficient and item factor loadings. Then, normality tests (Shapiro-Wilk test) and homogeneity of variance tests (Levene test) were performed for the older and younger groups. If data met both normality and homogeneity of variance criteria, independent sample 2-tailed *t* tests were used to analyze significant differences in overall flow experience and its dimensions between the 2 groups. If either criterion was not met, the nonparametric Wilcoxon rank-sum test was applied to examine significant differences in flow experience, with a significance level set at *α*=.05. Furthermore, due to the small sample size of this study, the corrected Cohen *d* (Hedges *g*) was used to calculate the effect size, and the 95% CI was estimated via the bootstrap method to enhance the robustness of the results. Posterior statistical power was calculated using G*Power (version 3.1.9.7; Heinrich-Heine-Universität Düsseldorf).

## Results

Participants took an average of 1.2 (SD 0.1) minutes to complete the questionnaire after the task. A total of 40 questionnaires were collected (100% response rate). Table 1 shows the demographic characteristics of the participants. Each participant completed all experimental tasks. The older and younger groups did not differ significantly in terms of sex distribution (*P*>.99), while, as expected, there was a significant difference in age between the 2 groups (*P*<.001).

**Table 1.** Participants’ demographic characteristics (N=40).

Characteristics	Older adults (n=21)	Younger adults (n=19)	*P* value
Age (y), mean (SD)	63.00 (6.64)	24.68 (1.16)	<.001
Sex, n (%)			>.99
Male	10 (48)	9 (47)	
Female	11 (52)	10 (53)	

**Figure 2.**
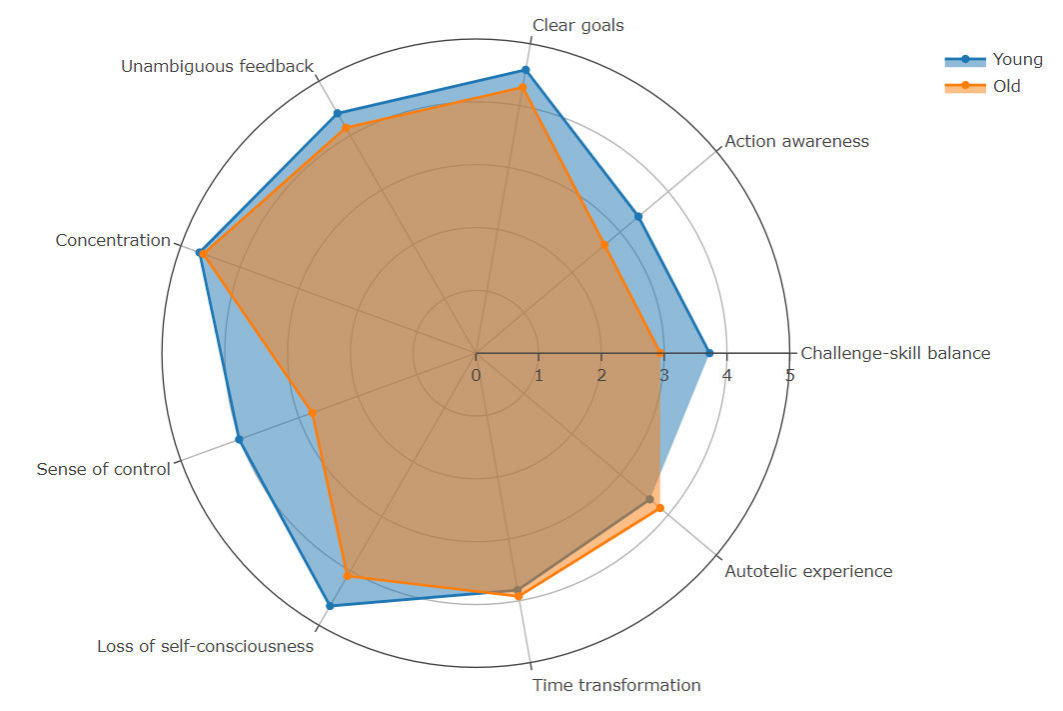
Radar chart of mean flow experience scores across 9 dimensions in older and younger adults.

The Cronbach α coefficient for the scale ranged from .83 to .98 (total scale *α*=.86), with item factor loadings between 0.44 and 0.98 (mean factor loading=0.87), indicating satisfactory internal consistency and cross-cultural, cross-domain applicability. Because the data did not conform to normal distribution or homogeneity of variance, we used the Wilcoxon rank-sum test (the nonparametric equivalent of the independent sample *t* test) for between-group comparisons (*α*=.05). The analysis revealed significant differences between the older and younger groups in overall flow experience and several dimensions (Table 2). A radar chart was plotted to further analyze the differences in the mean flow scores between the 2 groups (Figure 2), showing that younger adults scored higher than older adults in most dimensions (except for “time transformation” and “autotelic experience”).

**Table 2.** Age-related differences in flow experience dimensions (N=40; nonparametric Wilcoxon rank-sum test).

Dimensions	W value	*P* value	Cohen *d*	η^2^
Challenge-skill balance	339	<.001^a^	1.28	0.31
Action awareness	267	.07	0.73	0.13
Clear goals	271	.04^a^	0.65	0.10
Unambiguous feedback	256	.09	0.69	0.12
Concentration	182.5	.62	0.12	<0.01
Sense of control	389.5	<.001^a^	3.22	0.74
Loss of self-consciousness	268	.048^a^	0.57	0.08
Time transformation	224	.48	-0.12	<0.01
Autotelic experience	181.5	.63	-0.25	0.02
Total	339.5	<.001^a^	1.45	0.37

a *P*<.05.

**Figure 3.**
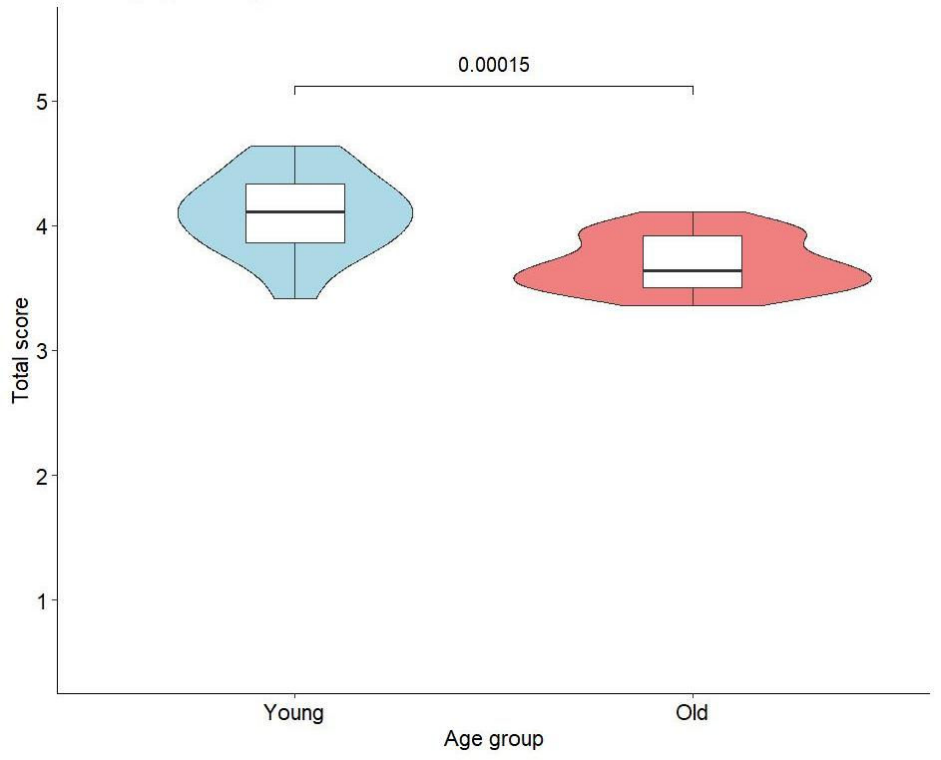
Violin plot of overall flow experience scores in older versus younger adults.

For overall flow experience, there was a significant difference between the older and younger groups (W=339.5; *P*<.001), indicating that older and younger adults obtain distinct flow experiences in the same BMCVG task, with the flow distribution among older adults being more concentrated (Figure 3). The effect size was large (Cohen *d*=1.45; η^2^=0.37), suggesting a substantial impact of age on overall flow experience. The corresponding statistical power reached 0.98, which also strengthened the reliability of the significant results.

**Figure 4.**
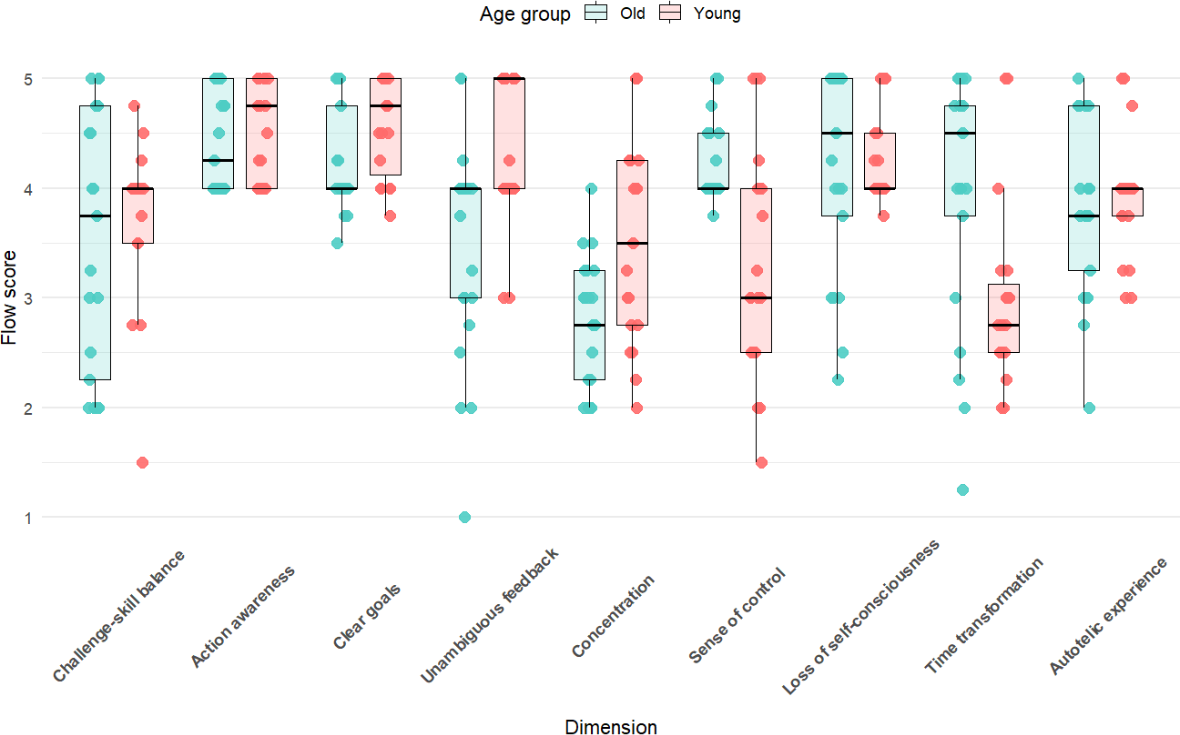
Comparison of flow experience scores across 9 dimensions between older and younger adults.

Regarding different flow dimensions (Figure 4), significant age differences were found in 4 dimensions: “challenge-skill balance” (W=339; *P*<.001; Cohen *d*=1.28; η^2^=0.31); “clear goals” (W=271; *P*=.04; Cohen *d*=0.65; η^2^=0.10); “sense of control” (W=389.5; *P*<.001; Cohen *d*=3.22; η^2^=0.74); and “loss of self-consciousness” (W=268; *P*=.048; Cohen *d*=0.57; η^2^=0.08). Among these, the “sense of control” dimension had the largest effect size (η^2^=0.74). Other dimensions such as “action awareness” (*P*=.07; Cohen *d*=0.73; η^2^=0.13) and “unambiguous feedback” (*P*=.09; Cohen *d*=0.69; η^2^=0.12) had *P* values close to the significance threshold, with effect size indicators suggesting a possible weak influence, but they did not reach statistical significance. No significant age differences were observed in “concentration” (*P*=.62; Cohen *d*=0.12; η^2^<0.01), “time transformation” (*P*=.48; Cohen *d*=−0.12; η^2^<0.01), and “autotelic experience” (*P*=.63; Cohen *d*=−0.25 η^2^=0.02), all of which had small effect sizes.

## Discussion

### Principal Findings

This study explored the impact of age on flow experience during BMCVG rehabilitation tasks. The results demonstrated that older adults scored significantly lower than younger adults in terms of overall flow experience and several core dimensions. Specifically, statistically significant differences were observed between the 2 groups in 4 dimensions: “challenge-skill balance” (*P*<.001; η^2^=0.31); “clear goals” (*P*=.04; η^2^=0.10); “sense of control” (*P*<.001; η^2^=0.74); and “loss of self-consciousness” (*P*=.048; η^2^=0.08). Among these dimensions, “sense of control” had the largest effect size, indicating that age differences had the most prominent impact on this dimension. These findings provide direct empirical evidence that age significantly shapes flow experience in BMCVG rehabilitation tasks.

### Comparison to Previous Work

The perceived match between task challenge and personal skill among older adults was significantly lower than that among younger adults (*P*<.001; η^2^=0.31), meaning older adults perceived task challenges as being either too high or too low for their own skills, resulting in a poor match between challenge and skill. This may be related to age-related cognitive decline. Meta-analyses have shown that physical activity levels have a protective effect on cognitive function [23-25]. Older adults generally experience a degradation of brain function due to reduced physical activity [26]. Especially when task challenges increase, the activation level of brain regions in older adults is equivalent to or lower than that in younger adults, with poorer behavioral performance [27]. Additionally, the complexity of motor tasks (such as those requiring high coordination) places more demanding cognitive requirements on older adults, leading to more obvious cognitive decline. We speculate that cognitive differences between older and younger adults when performing the same task may contribute to the differences in the “challenge-skill balance” dimension.

In the “clear goals” dimension, older adults scored significantly lower than younger adults (*P*=.04; η^2^=0.10), indicating they had more difficulty clearly perceiving task goals during the task. This is likely related to the decline in cognitive abilities and task comprehension in older adults. With age, individuals experience slower information processing and reduced attention maintenance, making it harder for them to quickly understand and internalize task goals. Research has shown that older adults process information more slowly, which may lead to poor performance in tasks requiring rapid target recognition and response [28]. Furthermore, older adults’ perception of task goals may be more influenced by situational factors, such as the complexity of visual information and the clarity of cues, which may further affect their goal perception in BMCVG tasks. Studies have shown that in visual search tasks, if the target presentation time is short or there are many interfering factors, older adults may have difficulty clearly perceiving the target [29].

Older adults had a significantly lower sense of control over the task than younger adults (*P*<.001; η^2^=0.74), meaning they were less likely to feel a sense of mastery over the task, and the difference in this dimension was the most significant. This result supports the findings of Hibbs et al [30], who found that older adults had a weaker sense of control than younger adults in low-screen virtual reality conditions, and this study further confirms the significance of this result. This may be related to the objective physical and psychological conditions of older adults. On the one hand, due to the decline in motor abilities, older adults may feel limited control over tasks that require rapid response or fine motor control, affecting their sense of task mastery. Research has shown that older adults exhibit reduced accuracy, increased variability, and prolonged execution time when performing bimanual motor tasks, failing to achieve expected performance [31]. On the other hand, older adults may experience more anxiety or lack confidence in task completion when faced with new tasks [32]. This decline in self-efficacy may affect their sense of control over tasks. Especially when faced with new or complex tasks, older adults may doubt their abilities, leading to a lower sense of control. As people enter old age, physical functions and activity levels gradually decline, and psychological characteristics also change [33]. Due to reduced sensory abilities, slower neuromotor speed, and memory decline, older adults become more cautious. We therefore propose that the decline in physical abilities and self-efficacy is the primary reason for the lower sense of control observed in older adults.

The “loss of self-consciousness” dimension showed that older adults scored significantly lower than younger adults (*P*=.048; η^2^=0.08), indicating they had more difficulty fully immersing themselves and experiencing self-forgetfulness in BMCVG tasks. This is consistent with the research by Boot et al [34] on age-related differences in immersion in virtual environments, which pointed out that many older adults have less experience and proficiency with emerging technologies, which may become a barrier to their integration into new virtual environments. In addition, research has shown that there are differences in the sense of immersion in BMCVGs among different age groups, which may be related to motor performance and subjective experience [30]. We hypothesize that inattention or memory loss in older adults may lead to poor motor performance and concerns about task failure or mistakes, making it difficult for them to fully engage in the BMCVG experience while maintaining a high level of self-awareness.

In summary, older adults’ lower scores in these dimensions may stem from declines in cognitive abilities, objective physical strength, and self-efficacy, while younger adults adapt more easily, maintaining immersion and task mastery. These findings highlight the need for age-sensitive rehabilitation task design. Designers should optimize task difficulty, provide clearer goals, and reduce motor demands, especially to enhance older adults’ sense of control and immersion.

It is worth noting that in this study, dimensions such as “action awareness” (*P*=.07; η^2^=0.13); “unambiguous feedback” (*P*=.09; η^2^=0.12); “concentration” (*P*=.62; η^2^<0.01); “time transformation” (*P*=.48; η^2^<0.01); and “autotelic experience” (*P*=.63; η^2^=0.02) did not show significant age differences. Among them, the first two were near the significance threshold (.05), and the effect sizes were close to medium levels. Although they did not reach significance, increasing the sample size in the future may further reveal potential differences. The low effect sizes in “concentration,” “time transformation,” and “autotelic experience” suggest that these flow characteristics may be more universal. The consistency in the “concentration” dimension may be related to the intuitiveness of the task design, where the task’s low cognitive load and situational attractiveness allow older adults to maintain high focus. The lack of differences in “time transformation” may be due to the short task duration (average 5 min), where differences in time perception between older and younger adults may not have been fully reflected. The nonsignificant difference in “autotelic experience” is consistent with previous research findings, reflecting the universality of the task’s interestingness design. Türksoy et al [13] pointed out that similar task interestingness can provide similar intrinsic motivation experiences. Therefore, the low effect sizes (η^2^<0.02) in “concentration,” “time transformation,” and “autotelic experience” are more likely to reflect true commonalities between age groups. For the “action awareness” (*P*=.07) and “unambiguous feedback” (*P*=.09) dimensions close to the significance threshold, their medium effect sizes (η^2^=0.12‐0.13) suggest that a larger sample size may be needed to confirm potential differences in these dimensions.

### Limitations

This study has several limitations. First, the use of a single BMCVG task limits the external validity of our findings. The task primarily involves limb coordination and reaction speed, and the observed age-related differences in flow might not be generalizable to other types of rehabilitation tasks, such as those focused on cognitive training, fine motor skills, or balance control. The task-dependent nature of flow experience means that different game genres could yield different results. Second, our sample was confined to younger and older adults, excluding a middle-aged cohort. This binary comparison prevents a full understanding of how flow experience evolves across the lifespan. Incorporating a middle-aged group would provide a more complete picture of the developmental continuum of flow in BMCVG environments. Furthermore, the recruitment of older adults from the Beihang University community and younger adults from the university’s student body poses another potential limitation. This specific sample may introduce a bias toward individuals with relatively higher education, cognitive function, and socioeconomic status, potentially affecting the generalizability of our results to a broader population with more diverse backgrounds. Third, while the gender distribution within our groups was relatively balanced, this study did not analyze potential gender-based differences in flow experience. Given that some research suggests gender can influence gaming preferences and experiences, this remains an area for future investigation. Finally, although the post hoc power analysis indicated adequate power for the primary outcome, such analyses depend on observed effect sizes and should not substitute for a priori sample size planning.

### Future Directions

To build upon our findings, future research should address these limitations. A key priority is to expand the variety of BMCVG rehabilitation tasks investigated. Studies could compare tasks with different cognitive and motor demands to determine whether the age-related differences in flow observed here are consistent across various rehabilitation scenarios. In addition, although the Xbox Kinect platform provided a validated and accessible tool for this study, future research should examine whether similar age-related flow differences are observed with newer motion-sensing technologies (eg, Kinect V2, depth cameras, and virtual reality systems). Such investigations would help establish the generalizability of our findings across different rehabilitation platforms and ensure technological relevance in evolving clinical and community settings. Furthermore, future studies should include a middle-aged demographic (eg, people aged between 30 and 55 y) to map the trajectory of flow experience across a broader age spectrum, moving beyond a simple young-versus-old comparison. This would allow for a more nuanced understanding of age-related changes. Finally, considering a wider range of individual differences, such as gender, personality traits, and educational level, could help tailor BMCVG rehabilitation designs to meet personalized user needs more effectively.

### Conclusions

This cross-sectional study (N=40) revealed a significant impact of age on flow experience in BMCVG rehabilitation tasks. Older adults exhibited lower flow levels than younger adults, especially in “challenge-skill balance,” “clear goals,” “sense of control,” and “loss of self-consciousness.” The largest effect was observed in the “sense of control” dimension (Cohen *d*=3.22). These findings indicate that age-related differences in cognitive and motor functions affect subjective experiences during BMCVG rehabilitation tasks. Designing BMCVG rehabilitation tasks for older adults should emphasize optimization of task difficulty, explicit goal cues, and reduced motor demands, thereby improving engagement and outcomes.

## Supplementary material

10.2196/76278 Multimedia Appendix 1 Flow State Scale-2 questionnaire.

## Abbreviations

BMCVG: body movement–controlled video game
CFSS-2: Chinese version of the Flow State Scale-2
FSS-2: Flow State Scale-2
