# Supplementary material for: Age Differences in Flow Experience During Body Movement–Controlled Video Game Rehabilitation Tasks: Cross-Sectional Study
Source: JMIR Serious Games. 2025 Oct 29;13:e76278. doi: 10.2196/76278 (PMC12571201; doi:10.2196/76278)
Supplement: Multimedia Appendix 1 [file games-v13-e76278-s001.pdf]

Flow State Scale-2

Please answer the following questions based on your experience during the task you just completed. These questions relate to the thoughts and experiences you may have encountered while participating in the activity. There are no right or wrong answers. Reflect on how you felt during the activity and select the number that best matches your experience and thoughts.

name：__________ Gender：_____ Age：_____		Strongly Disagree	Disagree	Neither Disagree nor Agree	Agree	Strongly Agree
1	I was challenged, but I believed my skills would allow me to meet the challenge.	1	2	3	4	5
2	I made the correct movements without thinking about trying to do so.	1	2	3	4	5
3	I knew clearly what I wanted to do.	1	2	3	4	5
4	It was really clear to me how my performance was going.	1	2	3	4	5
5	My attention was focused entirely on what I was doing.	1	2	3	4	5
6	I had a sense of control over what I was doing.	1	2	3	4	5
7	I was not concerned with what others may have been thinking of me.	1	2	3	4	5
8	Time seemed to alter(either slowed down or speeded up).	1	2	3	4	5
9	I really enjoyed the experience.	1	2	3	4	5
10	My abilities matched the high challenge of the situation.	1	2	3	4	5
11	Things just seemed to be happening automatically.	1	2	3	4	5
12	I had a strong sense of what I wanted to do.	1	2	3	4	5
13	I was aware of how well I was performing.	1	2	3	4	5
14	It was no effort to keep my mind on what was happening.	1	2	3	4	5
15	I felt like I could control what i was doing.	1	2	3	4	5
16	I was not concerned with how others may have been evaluating me.	1	2	3	4	5
17	The way time passed seemed to be different from normal.	1	2	3	4	5

18	I loved the feeling of the performance and want to capture it again.	1	2	3	4	5
19	I felt I was competent enough to meet the high demands of the situation.	1	2	3	4	5
20	I performed automatically, without thinking too much.	1	2	3	4	5
21	I knew what I wanted to achieve.	1	2	3	4	5
22	I had a good idea while I was performing about how well I was doing.	1	2	3	4	5
23	I had total concentration.	1	2	3	4	5
24	I had a feeling of total control.	1	2	3	4	5
25	I was not concerned with how I was presenting myself.	1	2	3	4	5
26	It felt like time went by quickly.	1	2	3	4	5
27	The experience left me feeling great.	1	2	3	4	5
28	The challenge and my skills were at an equally high level.	1	2	3	4	5
29	I did things spontaneously and automatically without having to think.	1	2	3	4	5
30	My goals were clearly defined.	1	2	3	4	5
31	I could tell by the way I was performing how well I was doing.	1	2	3	4	5
32	I was completely focused on the task at hand.	1	2	3	4	5
33	I felt in total control of my body.	1	2	3	4	5
34	I was not worried about what others may have been thinking of me.	1	2	3	4	5
35	I lost my normal awareness of time.	1	2	3	4	5
36	I found the experience extremely rewarding.	1	2	3	4	5

Jackson SA, Eklund RC. Assessing flow in physical activity: the Flow State Scale-2 and Dispositional Flow Scale-2. J Sport Exerc Psychol. 2002;24(2):133-150. [doi: 10.1123/jsep.24.2.133]
